# Stromal cell‐derived factor‐1/Exendin‐4 cotherapy facilitates the proliferation, migration and osteogenic differentiation of human periodontal ligament stem cells in vitro and promotes periodontal bone regeneration in vivo

**DOI:** 10.1111/cpr.12997

**Published:** 2021-01-29

**Authors:** Qianyu Liang, Lingqian Du, Rui Zhang, Wenyan Kang, Shaohua Ge

**Affiliations:** ^1^ Department of Periodontology School and Hospital of Stomatology Cheeloo College of Medicine Shandong University & Shandong Key Laboratory of Oral Tissue Regeneration & Shandong Engineering Laboratory for Dental Materials and Oral Tissue Regeneration Jinan Shandong China; ^2^ Department of Stomatology The Second Hospital Cheeloo College of Medicine Shandong University Jinan Shandong China; ^3^ Department of Endodontics Hospital of stomatology Zunyi Medical University Zunyi Guizhou China

**Keywords:** exendin‐4, osteogenic differentiation, periodontal bone regeneration, periodontal ligament stem cell, stromal cell‐derived factor‐1

## Abstract

**Objectives:**

Stromal cell‐derived factor‐1 (SDF‐1) actively directs endogenous cell homing. Exendin‐4 (EX‐4) promotes stem cell osteogenic differentiation. Studies revealed that EX‐4 strengthened SDF‐1‐mediated stem cell migration. However, the effects of SDF‐1 and EX‐4 on periodontal ligament stem cells (PDLSCs) and bone regeneration have not been investigated. In this study, we aimed to evaluate the effects of SDF‐1/EX‐4 cotherapy on PDLSCs in vitro and periodontal bone regeneration in vivo.

**Methods:**

Cell‐counting kit‐8 (CCK8), transwell assay, qRT‐PCR and western blot were used to determine the effects and mechanism of SDF‐1/EX‐4 cotherapy on PDLSCs in vitro. A rat periodontal bone defect model was developed to evaluate the effects of topical application of SDF‐1 and systemic injection of EX‐4 on endogenous cell recruitment, osteoclastogenesis and bone regeneration in vivo.

**Results:**

SDF‐1/EX‐4 cotherapy had additive effects on PDLSC proliferation, migration, alkaline phosphatase (ALP) activity, mineral deposition and osteogenesis‐related gene expression compared to SDF‐1 or EX‐4 in vitro. Pretreatment with ERK inhibitor U0126 blocked SDF‐1/EX‐4 cotherapy induced ERK signal activation and PDLSC proliferation. SDF‐1/EX‐4 cotherapy significantly promoted new bone formation, recruited more CXCR4^+^ cells and CD90^+^/CD34^‐^ stromal cells to the defects, enhanced early‐stage osteoclastogenesis and osteogenesis‐related markers expression in regenerated bone compared to control, SDF‐1 or EX‐4 in vivo.

**Conclusions:**

SDF‐1/EX‐4 cotherapy synergistically regulated PDLSC activities, promoted periodontal bone formation, thereby providing a new strategy for periodontal bone regeneration.

## Graphical abstract

Locally delivered stromal cell‐derived factor‐1 (SDF‐1) combined with systemically applied Exendin‐4 (EX‐4) synergestically recruited endogenous stem cells towards the rat periodontal defects. Further, the cotherapy enhanced the proliferation and osteogenic differentiation of the engrafted stem cells in coordination, thus significantly facilitated periodontal bone regeneration.

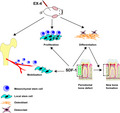

## 1 INTRODUCTION

Periodontitis is associated with many chronic diseases therefore represents a major global health problem.^1^ Present therapies for periodontitis such as subgingival scaling and root planning, can only remove inflammation and halt disease progression while failing to accomplish bone tissue reconstruction. Therefore, novel approaches are in high demands to achieve periodontal bone regeneration.^2^ Mesenchymal stem cells (MSCs) are regarded as the key element for tissue repair due to their potential to regenerate injured or pathologically damaged tissues and restore into a normal and healthy state.^3^ With the development of stem cell therapies, endogenous MSCs recruitment which harnesses the innate regenerative potential of the body suggests new effective therapeutic approaches.^4^

Chemokines are signalling molecules that can enhance cell migration, regulate immune responses and promote wound healing.^5^ Stromal cell‐derived factor‐1 (SDF‐1) belongs to the chemokine family, can promote the proliferation and migration of various MSCs in vitro by activating C‐X‐C chemokine receptor type 4 (CXCR4).^6^, ^7^, ^8^ In vivo experiments evidenced that topical application of SDF‐1 recruited MSCs to the wound area and promoted local vascular regeneration.^9^, ^10^ The defect area of periodontitis with insufficient cell number and poor cell activity is extremely unfavourable for subsequent periodontal bone regeneration. SDF‐1 could mobilize bone marrow mesenchymal stem cells (BMSCs) to periodontal defects by activating SDF‐1/CXCR4 signal axis.^7^ Mounting evidences demonstrated that the introduce of SDF‐1 enhanced the recruitment of endogenous cells to defects for regeneration.^10^, ^11^, ^12^ It is obvious that SDF‐1 is closely associated with the migration and growth of MSCs. However, SDF‐1 does not always enhance proper osteogenic differentiation of these cells.^13^ BMSCs transfected with SDF‐1 could only promote osteogenic differentiation of the cells in early‐stage, but not middle/late stage in vitro.^14^ The application of SDF‐1 alone is insufficient for favourable bone regeneration.^15^ The optimal method to potentiate periodontal bone regeneration is to recruit a sufficient number of endogenous stem cells by SDF‐1 and to promote committed differentiation by other growth factors or drugs.

Exendin‐4 (EX‐4), a full agonist of glucagon‐like peptide‐1 receptor (GLP‐1R), has the ability to promote insulin secretion and release, inhibit glucagon secretion, and delay gastric emptying.^16^ EX‐4 is widely used in clinical treatment of type 2 diabetes (T2DM) for its half‐life is longer than natural glucagon‐like peptide‐1 (GLP‐1).^17^ EX‐4 could control blood sugar, exert anti‐inflammatory, anti‐oxidant and protective effects on cardiomyocyte metabolism, and promote the proliferation and migration of MSCs.^18^, ^19^, ^20^, ^21^ Recently, studies have confirmed that EX‐4 has the capability to promote osteogenic differentiation, inhibit adipogenic differentiation of a variety of stem/precursor cells and promote bone formation to repair bone defects.^22^, ^23^, ^24^ More importantly, the number of CXCR4^+^ MSCs increased by EX‐4 and the PI3K/AKT‐SDF‐1/CXCR4 signalling pathway has been reported to play an important role in EX‐4‐mediated MSCs mobilization.^21^ Accordingly, both the recruitment effect of SDF‐1 and the osteogenic differentiation capability of MSCs could effectively be enhanced by EX‐4.^21^ Therefore, the application of SDF‐1 together with EX‐4 might be an effective strategy to augment periodontal bone regeneration.

The purpose of this study was to evaluate the effects of SDF‐1/EX‐4 cotherapy on the proliferation, migration and osteogenic differentiation of periodontal ligament stem cells (PDLSCs) in vitro. Afterwards, local application of SDF‐1 and systemic injection of EX‐4 were applied to a rat periodontal bone defect model. Endogenous cells recruitment, early osteolclasteogenesis and bone regeneration were evaluated to verify whether the cotherapy will provide a new strategic option for in situ periodontal bone regeneration.

## 2 MATERIALS AND METHODS

### 2.1 Cell culture

The study protocol was approved by the School and Hospital of Stomatology, Shandong University (Protocol Number: GR201603), and written informed consent was obtained. Human PDLSCs were isolated according to our previous study.^6^ Culture, osteogenic and adipogenic differentiation capabilities of human PDLSCs were performed as described in supplemental materials.

### 2.2 Cell proliferation assay

50 ng/mL SDF‐1 (Peprotech) and 10 nmol/L EX‐4 (R&D systems) were selected for the subsequent experiments according to our supplementary materials. PDLSCs were seeded in 96‐well plates at a density of 3 × 10^3^ cells/well and cultured in basic media [Dulbecco's modified Eagle's medium (DMEM) with 10% foetal bovine serum (FBS)]. After 24 hours, cells were cultured in maintenance media (DMEM with 2% FBS) or maintenance media with SDF‐1, EX‐4, or SDF‐1+EX‐4 for 1, 3 or 5 days. Cell‐counting kit‐8 (CCK8, Dojindo Laboratories) solution was added. The absorbance was detected by a microplate reader (SPECTROstar Nano, BMG Labtech) at a wavelength of 450 nm. Experiments were performed in sextuplicate (N = 6).

### 2.3 Cell migration assay

The migratory effect of SDF‐1 and EX‐4 on PDLSCs was evaluated by an 8 µm transwell chamber (Corning). PDLSCs were seeded onto the upper chamber at a density of 5 × 10^4^ cells/well and cultured in maintenance media. The lower plates were supplemented in 500 µL maintenance media with SDF‐1, EX‐4, or SDF‐1+EX‐4. 500 µL maintenance media served as a negative control (NC) and 500 µL basic media served as a positive control (PC). After 20 hours, cells that had migrated through the membrane were fixed with 4% paraformaldehyde (Sigma Aldrich) and stained with 0.1% crystal violet (Solarbio). Then cells were observed under a microscope and six randomly selected high‐power microscopic fields (×200) per filter were counted. Experiments were performed in triplicate (N = 3).

### 2.4 Cell osteogenic differentiation assay

PDLSCs were seeded in 6‐well plates at a density of 2 × 10^5^ cells/well and cultured in osteogenic inductive media [OM, basic media with 10^−8^ mol/L dexamethasone (Solarbio), 50 mg/L ascorbic acid and 10 mmol/L β‐glycerophosphate (Sigma Aldrich)], OM with SDF‐1, EX‐4, or SDF‐1+EX‐4. Experiments were performed in sextuplicate (N = 6).

#### 2.4.1 Alizarin Red S staining

After 21‐day induction, PDLSCs were fixed with 4% paraformaldehyde and extracellular matrix calcification was estimated by 2% Alizarin Red S (pH 4.3, Sigma Aldrich). 10% (w/v) cetylpyridinium chloride (CPC, Solarbio) and 10 mmol/L sodium phosphate solution were used to quantify the relative amount of calcium. The absorbance was measured at 562 nm wavelength.

#### 2.4.2 Alkaline phosphatase activity assay

After 7, 14 days induction, PDLSCs were lysed with 1% Triton‐X (Solarbio) for 30 minutes. Protein concentration of collected cell lysates was measured by BCA assay (Solarbio). Alkaline phosphatase (ALP) activity in these lysates was detected according to assay kit (Nanjing Jiancheng Bioengineering Institute). The absorbance was measured at 520 nm wavelength.

#### 2.4.3 RNA isolation & quantitative real‐time polymerase chain reaction (qRT‐PCR) analysis of osteogenesis‐related gene expression

After induction for 7, 14 and 21 days, total RNA was extracted with Trizol® (Takara). 1 μg mRNA was reverse‐transcribed to cDNA using PrimeScript™ RT reagent Kit with gDNA Eraser (Takara). Then qRT‐PCR was performed with SYBR® Premix Ex Taq II (Tli RNaseH Plus; Takara) on Light Cycler Roche 480 II Real‐Time PCR System (Roche). Glyceraldehyde‐3‐phosphate dehydrogenase (GAPDH) primer was used as a housekeeping gene and data were analysed using the 2^(−ΔΔCt)^ method. The sequences of the primers of *GAPDH, ALP*, *osteopontin* (*OPN*), *bone sialoprotein* (*BSP*) and *osteocalcin* (*OCN*) were shown in Table 1.

**TABLE 1:** Primers sequences for quantitative real‐time polymerase chain reaction (qRT‐PCR)

Gene	Primer sequences	
	5′ ~ 3′Forward	5′ ~ 3′Reverse
*GAPDH*	GCACCGTCAAGGCTGAGAAC	TGGTGAAGACGCCAGTGGA
*ALP*	ATGGGATGGGTGTCTCCACA	CCACGAAGGGGAACTTGTC
*OPN*	TCCTAGCCCCACAGACCCTT	CACACTATCACCTCGGCCAT
*BSP*	CCCCACCTTTTGGGAAAACCA	TCCCCGTTCTCACTTTCATAGAT
*OCN*	TCACACTCCTCGCCCTATT	GATGTGGTCAGCCAACTCG

Abbreviations: ALP: alkaline phosphatase; BSP: bone sialoprotein; GAPDH: glyceraldehyde‐3‐phosphate dehydrogenase; OCN: osteocalcin; OPN: osteopontin.

### 2.5 Western blotting assay

Cells were seeded in 6‐well plates at a density of 3 × 10^5^ cells/well and cultured in basic media. To examine the effect of SDF‐1 and EX‐4 cotherapy on extracellular signal‐regulated kinase (ERK), cells were cultured with basic media, SDF‐1, EX‐4, or SDF‐1+EX‐4 for 2 minutes. To determine whether the specific ERK inhibitor U0126 (MedChemExpress) influenced the ERK phosphorylation, cells were cultured with basic media, SDF‐1+EX‐4, SDF‐1+EX‐4+U0126 or U0126 for 2 minutes. Cells were then lysed with RIPA (Solarbio) containing 1% phosphatase inhibitor (CoWin Biosciences) and 1% protease inhibitor (Solarbio). The collected proteins were loaded onto 10% sodium dodecyl sulphate‐polyacrylamide gel electrophoresis (SDS‐PAGE) gels and then transferred to polyvinylidene fluoride (PVDF) membranes (Millipore). The membranes were blocked with 5% non‐fat dry milk and incubated with anti‐GAPDH antibody (1:10 000, No. 10494‐1‐AP, Proteintech), anti‐total‐ERK antibody (1:1000, 4695, Cell Signaling Technology), or anti‐phospho‐ERK antibody (1:2000, 4370, Cell Signaling Technology) overnight at 4°C. The immunoreactive bands on membranes were incubated with horseradish peroxidase‐labelled secondary antibodies (1:10 000, SA00001‐2, Proteintech) for 1 hour and visualized with chemiluminescence system (Millpore) and analysed with image j 1.44 software (NIH). Experiments were performed in triplicate (N = 3).

### 2.6 Effect of ERK inhibitor U0126 on SDF‐1 and EX‐4 mediated PDLSC proliferation

A specific ERK inhibitor U0126 was used to clarify the role of ERK signalling pathway in SDF‐1+EX‐4 mediated PDLSC proliferation. Briefly, PDLSCs were seeded in 96‐well plates and cultured in basic media. After 24 hours, cells were cultured in maintenance media or maintenance media with SDF‐1+EX‐4, SDF‐1+EX‐4+U0126 or U0126 for 1, 3 or 5 days. Cell proliferation was quantified using a CCK8 kit as described above. Experiments were performed in sextuplicate (N = 6).

### 2.7 Preparation of animal model

#### 2.7.1 Preparation of collagen membranes

Medical collagen membranes (Zhenghai Biotechnology) were cut into small pieces, placed in 96‐well plates and cultured with 100 μL SDF‐1 (50 μg/mL) or phosphate‐buffered saline (PBS, Hyclone) according to our previous study.^11^ The soaked collagen scaffolds were incubated at 4°C overnight before grafting.

#### 2.7.2 Preparation of periodontal defect model

The experimental procedure was approved by the Institutional Review Board, Hospital of Stomatology, Zunyi Medical University (Protocol Number: ZYKQ‐IRB‐KY‐2019‐002). Sixty 8‐week‐old adult Wistar rats (200‐250 g, male) were used in this study. Periodontal defects were prepared on bilateral mandibles of each rat. The rats were randomly divided into two groups. Thirty rats intraperitoneal injected daily with 0.9% sodium chloride solution, the membrane loaded with PBS implanted into the left side defect was control group, while the membrane loaded with SDF‐1 implanted into the right side was SDF‐1 group. Thirty rats intraperitoneal injected daily with EX‐4, the membrane loaded with PBS implanted into the left side defect was EX‐4 group, while the membrane loaded with SDF‐1 implanted into the right side was SDF‐1+EX‐4 group. Six rats of each group were sacrificed respectively under general anaesthesia at 3 days, 1, 2, 4 and 8 weeks after surgery. Surgical procedures were performed as previously described.^11^ Briefly, after general anaesthesia, we exposed the mandible surface of the rat and removed the buccal periodontal support tissues of the mandibular first molar roots. The periodontal fenestration defects (length × width × depth: 5 mm × 4 mm × 1 mm) located 1 mm below the crest of the alveolar bone and 1 mm behind the front of the mandible were prepared. Collagen membranes loaded with SDF‐1 or PBS were implanted into the defect. The masseter muscle, skin was reset and sutured. Penicillin sodium (160 000 IU/mL) was administered daily for 5 days after surgery. The rats were sacrificed under general anaesthesia at 3 days, 1, 2, 4 and 8 weeks after surgery, and fixed with 4% paraformaldehyde solution by cardiac perfusion. The mandibles were harvested for the following experiments.

### 2.8 Micro‐CT analysis

The samples of mandibles were scanned in three dimensions (3D) by a micro‐CT scanner (PerkinElmer). The effective voxel size was 50 μm in high‐resolution mode. The results were performed 3D reconstruction using Quantum GX micro‐CT software (PerkinElmer). Bone volume/tissue volume (BV/TV), bone surface area/tissue volume (BS/TV), trabecular thickness (Tb.Th) and trabecular separation (Tb.Sp) were analysed by 3d slicer software package (Version 4.8.1, Surgical Planning Laboratory).

### 2.9 Histological analysis

The bilateral mandibles of each rat were decalcified with 10% ethylenediaminetetraacetate (EDTA, Solarbio), dehydrated with a series of graded ethanol solutions and embedded in paraffin wax. The embedded mandibles were cut in a buccal‐lingual direction to acquire transverse sections (5 μm thick). The prepared sections were stained with haematoxylin and eosin (H&E, Solarbio). The samples were observed under a BX53 microscope (Olympus Corporation) and measured with Image pro‐plus 6.0 Software (Media Cybernetics).

### 2.10 Immunofluorescence staining

The mandible sections were incubated with antigen retrieval solution (Solarbio), blocked with goat serum (Solarbio) and incubated with primary antibodies at 4°C overnight: rabbit anti‐CXCR4 antibody (1:200, ab124824, Abcam), rabbit anit‐CD34 antibody (1:200, ab81289, Abcam) and mouse monoclonal anti‐CD90 antibody (1:400, ab225, Abcam) for a double immunofluorescence staining of CD90/CD34. Goat anti‐rabbit IgG (1:200, SA00013‐4, Proteintech) or goat anti‐mouse IgG (1:200, SA00013‐1, Proteintech) was used as the secondary antibodies. Nuclei were visualized with 2‐(4‐amidinophenyl)‐6‐indolecarbamidine dihydrochloride (DAPI, Solarbio) and mounted. The images were photographed under a fluorescence microscope in the dark room.

### 2.11 TRAP staining

Sections were stained with a leucocyte acid phosphatase kit (Solarbio) according to the manufacturer's instructions, nuclei were visualized with methyl green. The number of TRAP^+^ cells was counted and measured by Image pro‐plus 6.0 software.

### 2.12 Immunohistochemical staining

The blocked sections were incubated with rabbit anti‐ALP antibody (1:100, ab95462, Abcam) or rabbit anti‐Collagen I (Col I) antibody (1:200, ab34710, Abcam) at 4°C overnight. Goat anti‐rabbit IgG H&L (horseradish peroxidase; 1:100, ab6721, Abcam) was used as the secondary antibody. Immunoreactions were detected with 3,3′‐diaminobenzidine (DAB, Solarbio) solution. Nuclei were visualized with haematoxylin and mounted. The images were photographed under a microscope. The mean optical density (OD) of ALP and Col I staining was measured by image pro‐plus 6.0 software.

### 2.13 Statistical analysis

All data were expressed as mean ± standard deviation (SD) and performed at least triplicates. Tests were analysed using graphpad prism software (version 6, MacKiev Software) and differences among more than two groups were analysed by one‐way or two‐way analysis of variance followed by Tukey's honestly significant difference comparison test. Value of *P* < .05 was defined as significant.

## 3 RESULTS

### 3.1 SDF‐1/EX‐4 cotherapy promoted the proliferation and migration of PDLSCs

#### 3.1.1 SDF‐1/EX‐4 cotherapy promoted the proliferation of PDLSCs

**FIGURE 1:**
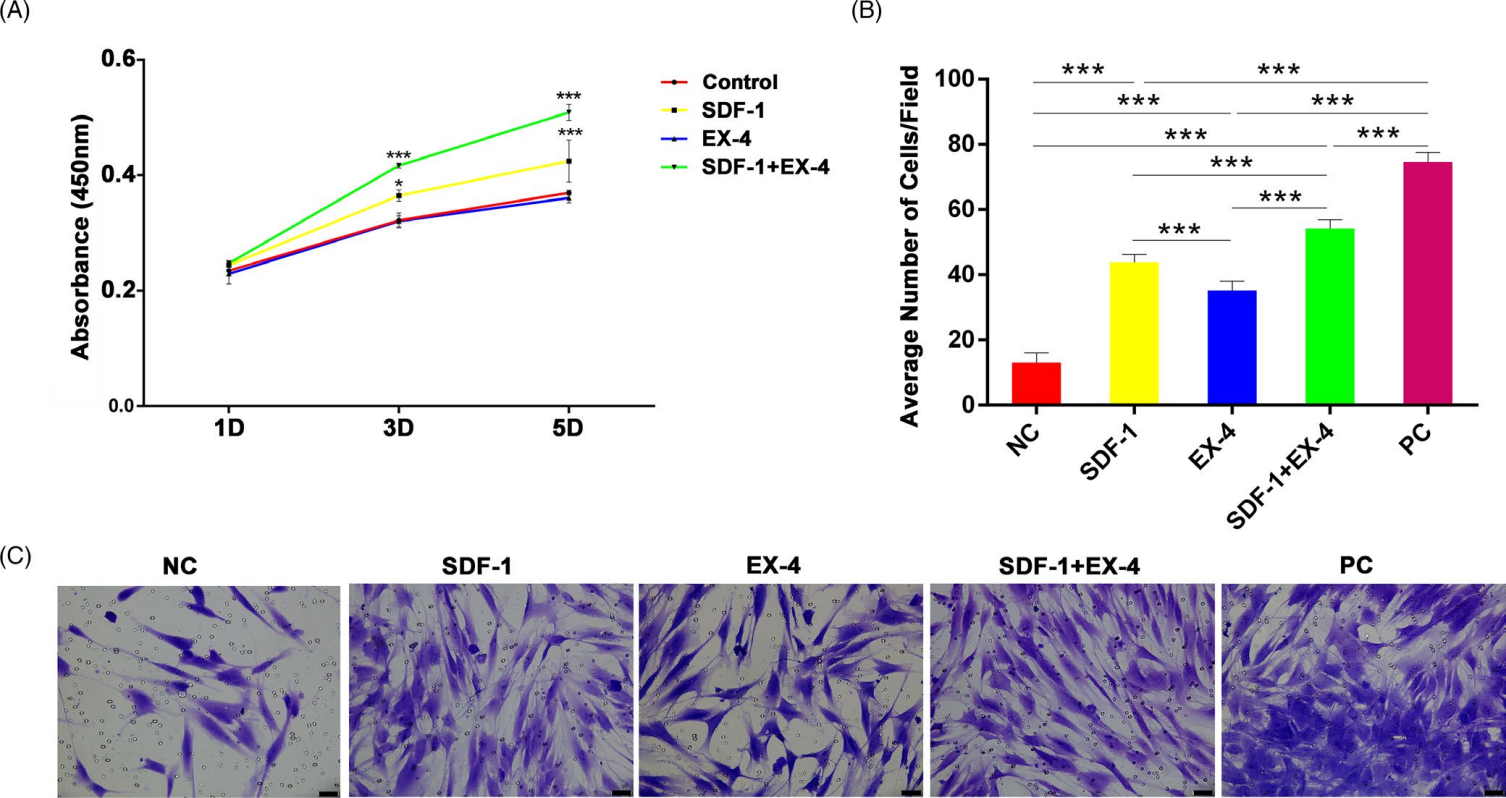
SDF‐1/EX‐4 cotherapy enhanced the proliferation and migration of PDLSCs. A, CCK8 assay showed SDF‐1+EX‐4 significantly promoted the proliferation of PDLSCs compared with control, SDF‐1 or EX‐4 groups. B, The average number of migrated cells in each microscope field in different groups. SDF‐1+EX‐4 significantly promoted the migration of PDLSCs compared with NC, SDF‐1 or EX‐4 groups. C, Crystal violet staining showed the cells that migrated to the undersurface of the membrane in different groups (×200). Scale bar: 50 μm. NC, negative control. PC, positive control. ^*^ *P* < .05 and ^***^ *P* < .001

SDF‐1/EX‐4 cotherapy significantly promoted the proliferation of PDLSCs compared with control, SDF‐1 or EX‐4 groups at day 3 and 5 (*P* < .001; Figure 1A). SDF‐1 significantly enhanced the proliferation of PDLSCs compared with control group at day 3 and 5 (*P* < .05). Meanwhile, EX‐4 presented no effect on PDLSC proliferation compared with control group (*P* > .05). The results showed that SDF‐1/EX‐4 cotherapy dramatically enhanced the proliferation of PDLSCs.

#### 3.1.2 SDF‐1/EX‐4 cotherapy facilitated the migration of PDLSCs

SDF‐1/EX‐4 cotherapy significantly enhanced the migration capacity of PDLSCs compared with NC, SDF‐1 or EX‐4 groups (54.2 ± 2.71 vs 13.0 ± 3.10, 43.8 ± 2.48, 35.2 ± 2.86 cells/field, *P* < .001; Figure 1B,C). Obviously, recruited cells in SDF‐1+EX‐4 group were less than PC (54.2 ± 2.71 vs 74.7 ± 2.88 cells/field, *P* < .001). SDF‐1 or EX‐4 also promoted PDLSC migration compared with NC (*P* < .001), and the promotion effect of SDF‐1 on cell migration was stronger than that in EX‐4 group (*P* < .001). These results indicated that SDF‐1/EX‐4 cotherapy enhanced PDLSC migration.

### 3.2 SDF‐1/EX‐4 cotherapy significantly promoted osteogenic differentiation of PDLSCs

**FIGURE 2:**
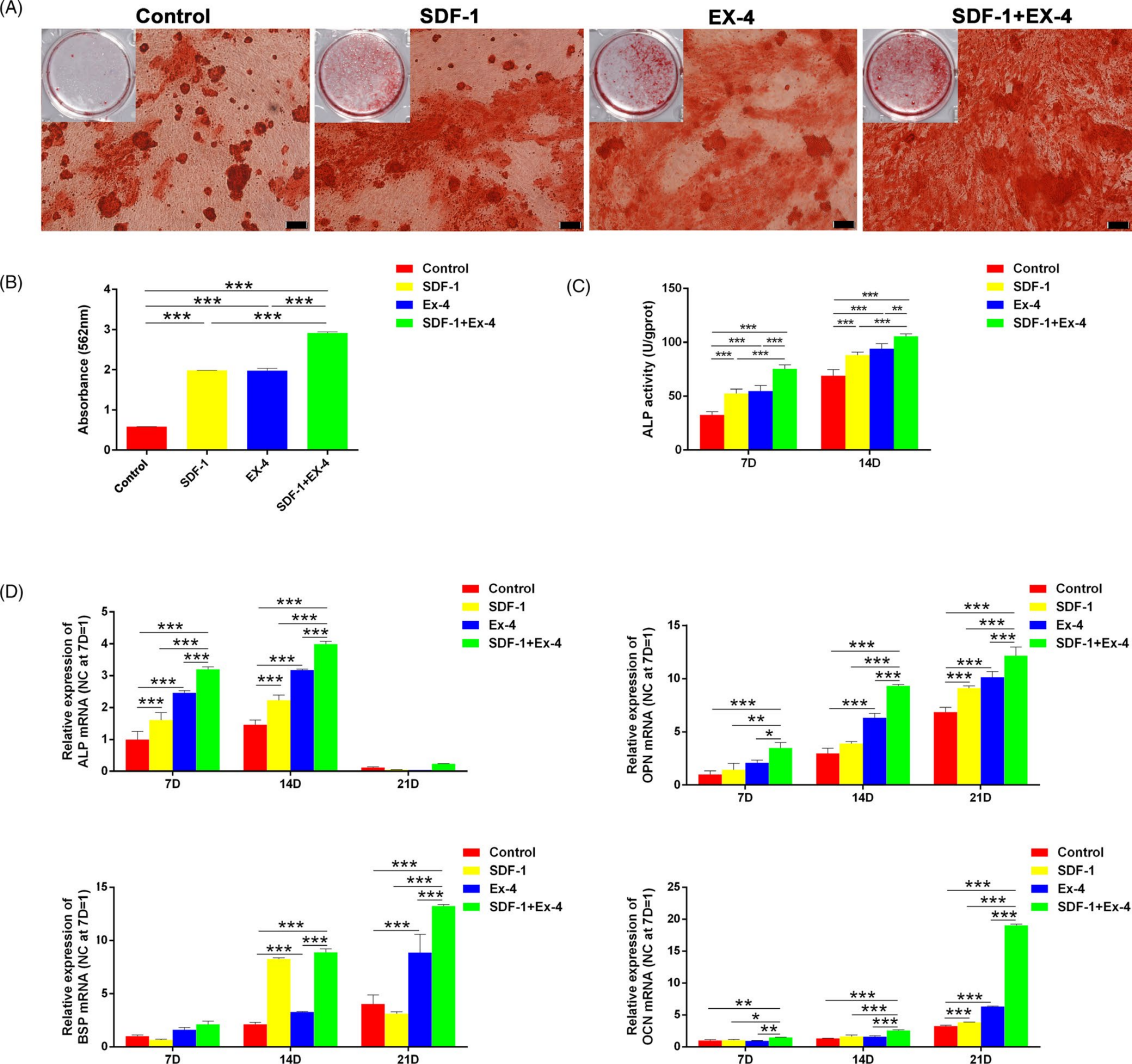
SDF‐1/EX‐4 cotherapy significantly promoted the formation of mineral deposition, ALP activity and gene expression of osteogenesis‐related markers. A, Representative photographs of Alizarin Red S staining in different groups at day 21 (×100). Scale bar: 100 μm. B, SDF‐1+EX‐4 significantly enhanced the formation of mineral deposition compared with control, SDF‐1 or EX‐4 groups. C, SDF‐1+EX‐4 significantly upregulated ALP activity compared with control, SDF‐1 or EX‐4 groups at day 7 and 14. D, Gene expression levels of *ALP*, *OPN*, *BSP* and *OCN* at day 7, 14 and 21. ^*^ *P* < .05, ^**^ *P* < .01 and ^***^ *P* < .001

At day 21, extracellular matrix calcification was detected by Alizarin Red S staining (Figure 2A) and the relative amount of calcium was quantified (Figure 2B). SDF‐1, EX‐4 or SDF‐1+EX‐4 significantly enhanced the formation of mineral deposition in comparison with OM (*P* < .001). SDF‐1+EX‐4 exhibited the maximal effect compared with the other three groups (*P* < .001). Unsurprisingly, SDF‐1, EX‐4 or SDF‐1+EX‐4 significantly upregulated ALP activity in comparison with OM at day 7 or 14 (*P* < .001; Figure 2C). SDF‐1+EX‐4 optimized the effect compared with the other three groups (*P* < .01). Gene expression levels of *ALP*, *OPN*, *BSP* and *OCN* were significantly upregulated in SDF‐1+EX‐4 group when compared with the other three groups (*P* < .05; Figure 2D). ALP increased dramatically in SDF‐1+EX‐4 group from day 7, and maximized at day 14. Gene expression levels of *OPN*, *BSP* and *OCN* increased over time, and the maximal levels exhibited at day 21.

### 3.3 SDF‐1/EX‐4 cotherapy activated ERK signalling pathway

**FIGURE 3:**
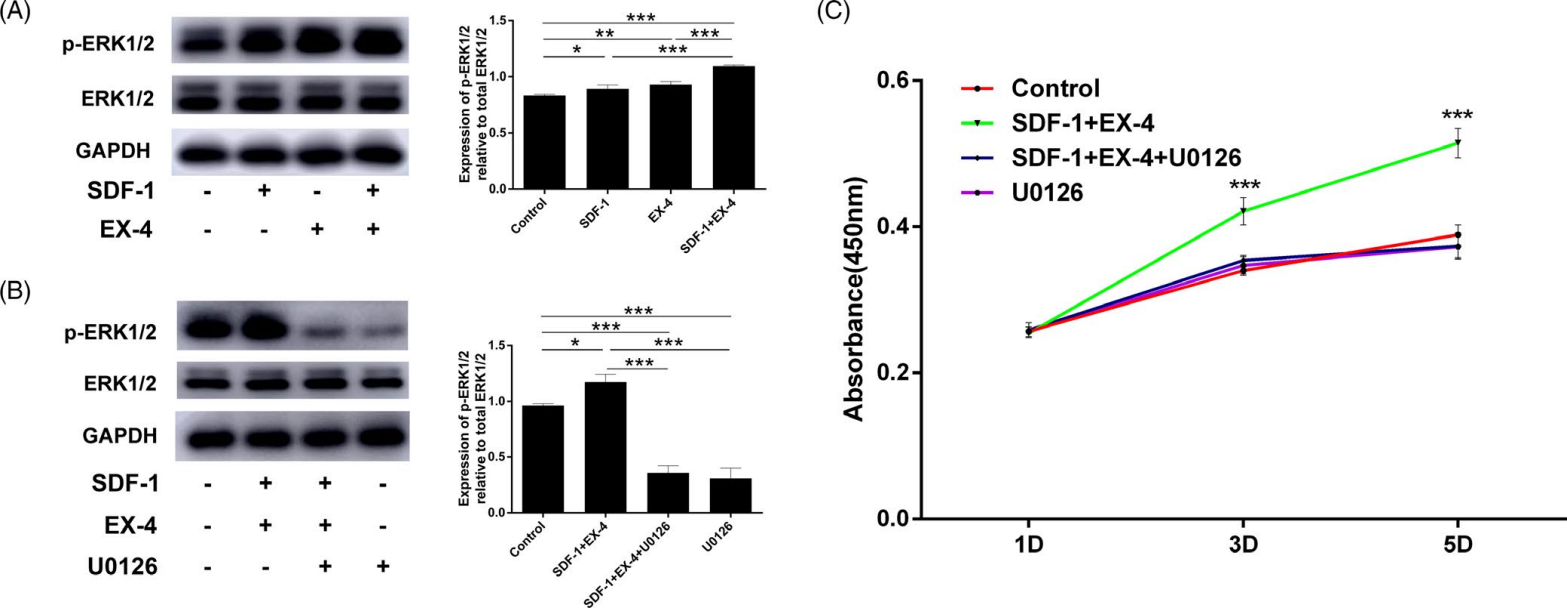
ERK inhibitor U0126 blocked SDF‐1/EX‐4 cotherapy induced ERK signal activation and PDLSC proliferation. A, SDF‐1+EX‐4 significantly promoted the phosphorylation of ERK compared with control, SDF‐1 or EX‐4 groups. B, Pretreatment of PDLSCs with U0126 blocked SDF‐1/EX‐4 cotherapy induced ERK activation. C, SDF‐1 cotherapy with EX‐4‐induced proliferation was inhibited by U0126. ^*^ *P* < .05, ^**^ *P* < .01 and ^***^ *P* < .001

ERK phosphorylation was significantly enhanced by SDF‐1 or EX‐4 compared with control group (*P* < .05; Figure 3A). Moreover, SDF‐1+EX‐4 significantly promoted ERK phosphorylation in comparison with the other three groups (*P* < .001). Further, ERK signalling pathway was blocked with an inhibitor, U0126. SDF‐1/EX‐4 cotherapy promoted ERK phosphorylation was significantly inhibited by U0126 (*P* < .001; Figure 3B). SDF‐1 cotherapy with EX‐4‐induced PDLSC proliferation was inhibited by U0126 (*P* < .001; Figure 3C).

### 3.4 3D digital micro‐CT assessment of bone formation

**FIGURE 4:**
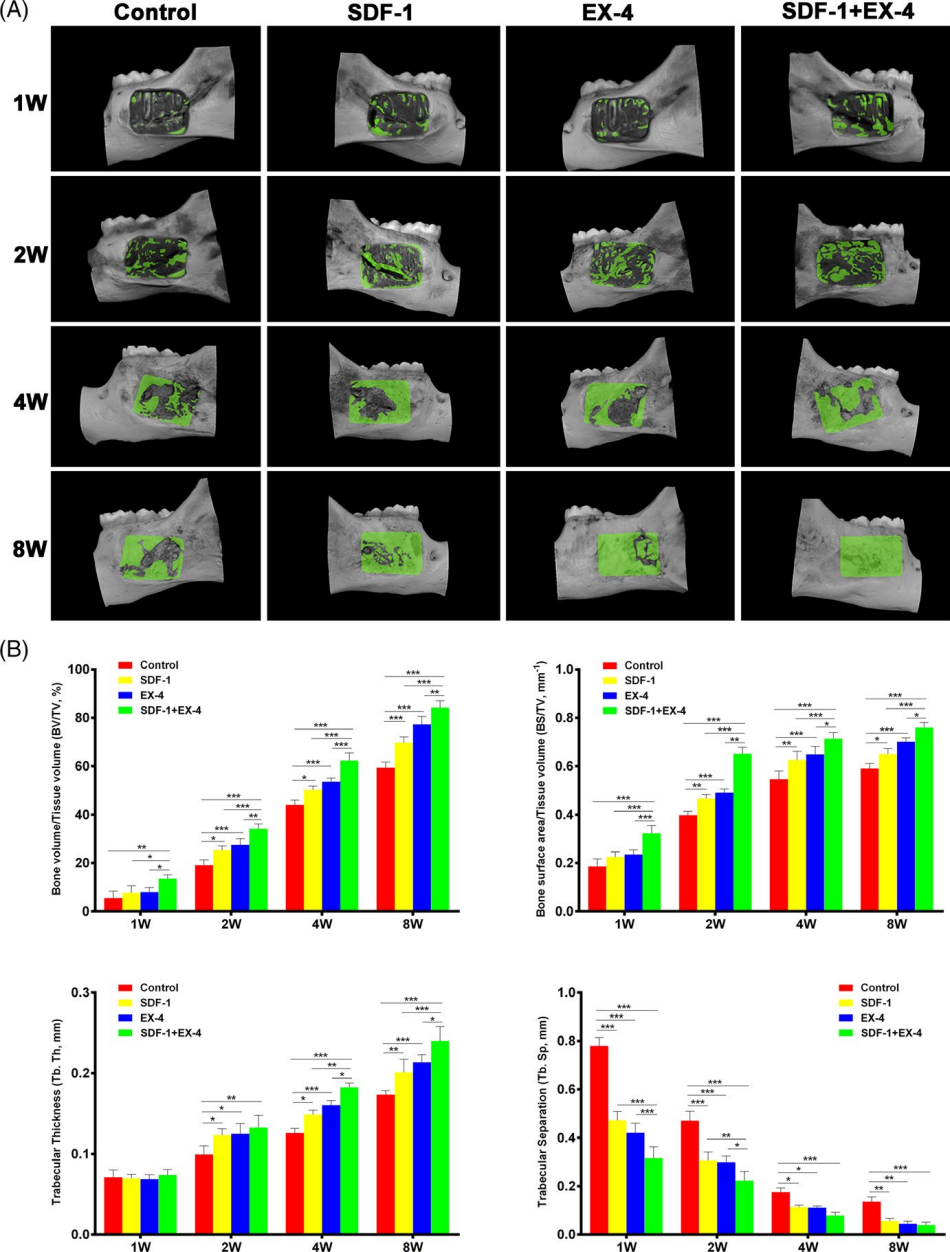
SDF‐1/EX‐4 cotherapy significantly enhanced the quantity and quality of newly formed bone at week 1, 2, 4 and 8 post‐surgery. A, Micro‐CT 3D reconstruction of four groups. B, The index of BV/TV, BS/TV, Tb.Th and Tb.Sp BV/TV, bone volume/tissue volume. BS/TV, bone surface area/tissue volume. Tb.Th, trabecular thickness. Tb.Sp, trabecular separation. ^*^ *P* < .05, ^**^ *P* < .01 and ^***^ *P* < .001

3D digital reconstructed micro‐CT images indicated SDF‐1+EX‐4 significantly promoted new bone formation compared with control, SDF‐1 or EX‐4 groups (Figure 4A). The newly formed bone in SDF‐1 or EX‐4 groups was significantly greater than that in control group. SDF‐1+EX‐4 group exhibited higher indexes of BV/TV, BS/TV and Tb.Th than control, SDF‐1 or EX‐4 groups (*P* < .05; Figure 4B). The cotherapy group displayed the highest indexes of BV/TV and BS/TV at each time point, indicating more new bones formed (*P* < .05). Moreover, SDF‐1+EX‐4 group significantly increased the index of Tb.Th at week 2, 4 and 8 (*P* < .05), indicating that the newly formed bones were denser. Tb.Sp in all groups were in a downtrend during the healing process. SDF‐1+EX‐4 group had faster descent rate than the other three groups at week 1 and 2 (*P* < .05), illustrating that new bone formed and matured earlier.

### 3.5 SDF‐1/EX‐4 cotherapy enhanced periodontal bone regeneration

**FIGURE 5:**
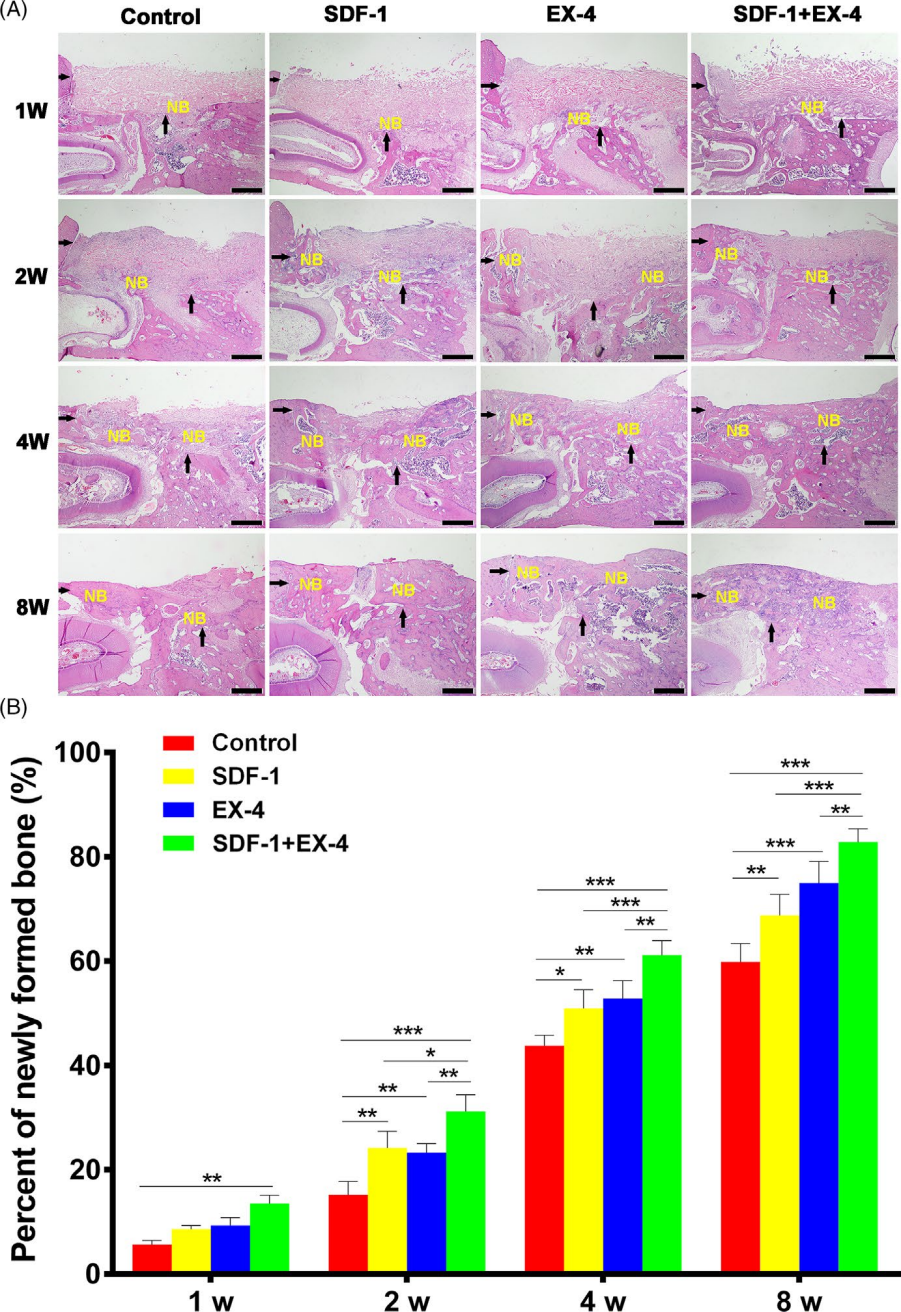
SDF‐1/EX‐4 cotherapy significantly promoted new bone formation. A, H&E staining of the mandible sections at week 1, 2, 4 and 8 post‐surgery (×40). Scale bar: 500 μm. Black arrows, edge of periodontal defect. NB, new bone. B, The percentage of newly formed bone at each time point in four groups. ^*^ *P* < .05, ^**^ *P* < .01 and ^***^ *P* < .001

Periodontal bone repair was assessed by H&E staining and histomorphometric measurements at week 1, 2, 4 and 8 post‐surgery (Figure 5A, B). SDF‐1+EX‐4 group formed more new bones than the other three groups at all time points (*P* < .05). SDF‐1 or EX‐4 also formed more new bones than control group (*P* < .05). Almost no new bone formed in the defect area of control group, and a small amount of new bone formed on the edge of defects in the other three groups at week 1 (*P* < .01). Newly formed bones could be observed in control group at week 2, but the percentage of newly formed bones was significantly less than the other three groups (*P* < .05), and the difference continued to the experimental end point. At week 4, the defect area of SDF‐1+EX‐4 group was filled with new bones, and the newly formed bones were similar to normal bone tissues in SDF‐1+EX‐4 group at week 8, while less bone and immature bone trabeculae formed in control group.

### 3.6 SDF‐1/EX‐4 cotherapy promoted the engraftment of CXCR4^+^ cells and CD90^+^/CD34^−^ stromal cells in vivo

**FIGURE 6:**
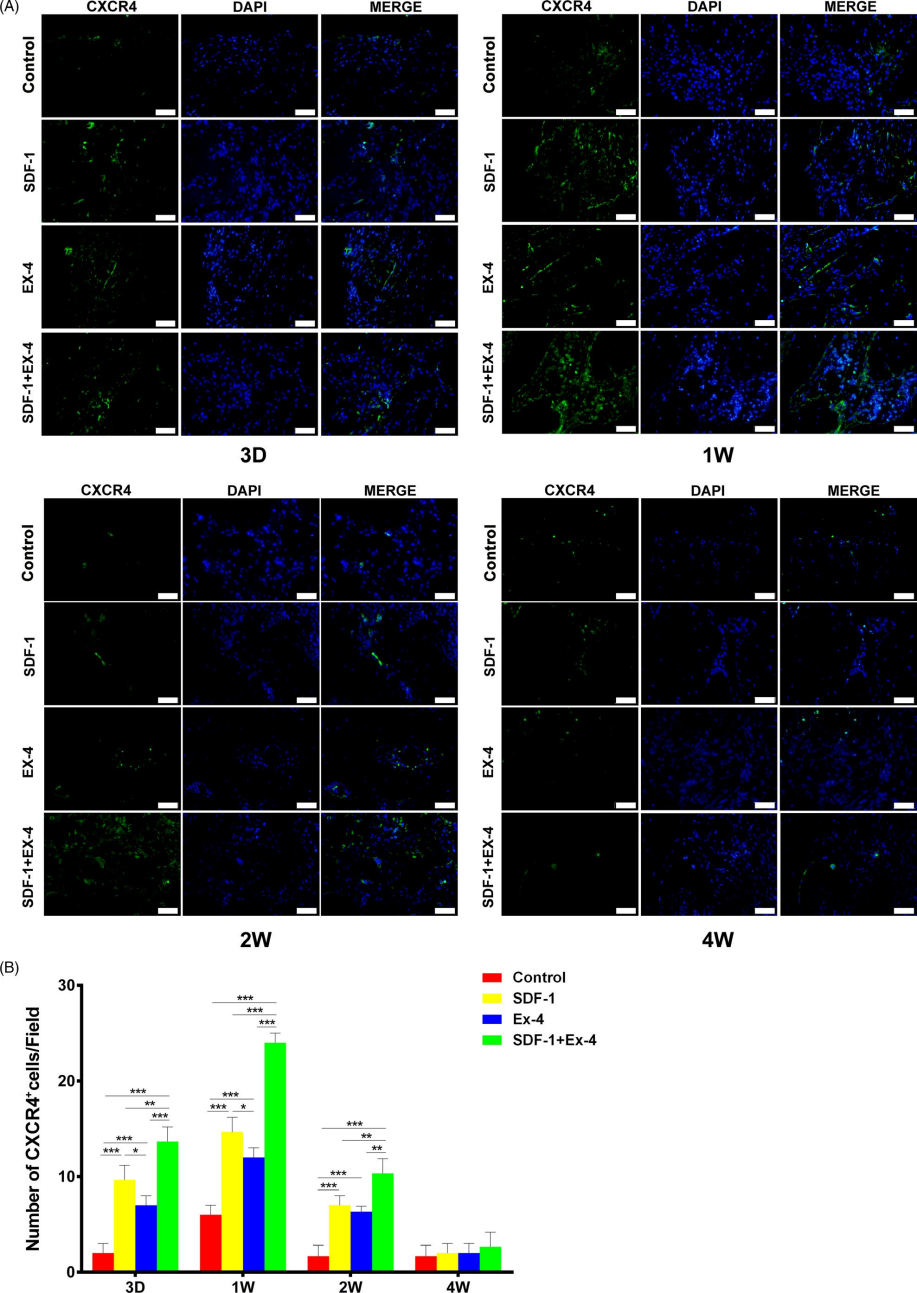
SDF‐1/EX‐4 cotherapy significantly promoted the migration of CXCR4^+^ cells in defect areas. A, Immunofluorescence staining of CXCR4 (green) in the mandible sections at day 3, week 1, 2 and 4 post‐surgery (×400). Scale bar: 50 μm. B, The number of CXCR4^+^ cells in SDF‐1+EX‐4 group was significantly larger than that in control, SDF‐1 or EX‐4 groups. The number of CXCR4^+^ cells peaked at week 1, reduced at week 2 and hardly detected at week 4. ^*^ *P* < .05, ^**^ *P* < .01 and ^***^ *P* < .001

The number of CXCR4^+^ cells in SDF‐1, EX‐4 or SDF‐1+EX‐4 groups was significantly larger than control group during the early stage of defect repair (day 3, week 1 and 2; *P* < .001; Figure 6A,B). Dramatically more CXCR4^+^ cells were observed in SDF‐1+EX‐4 group than that in SDF‐1 or EX‐4 groups at day 3 or week 1 (*P* < .01), and the promotion effect of SDF‐1 on CXCR4^+^ cell engraftment was stronger than that in EX‐4 group (*P* < .05). CXCR4^+^ cells decreased obviously at week 2, though the number of CXCR4^+^ cells in SDF‐1+EX‐4 group was still the largest (*P* < .01). At week 4, CXCR4^+^ cells in defect areas were hardly detected.

**FIGURE 7:**
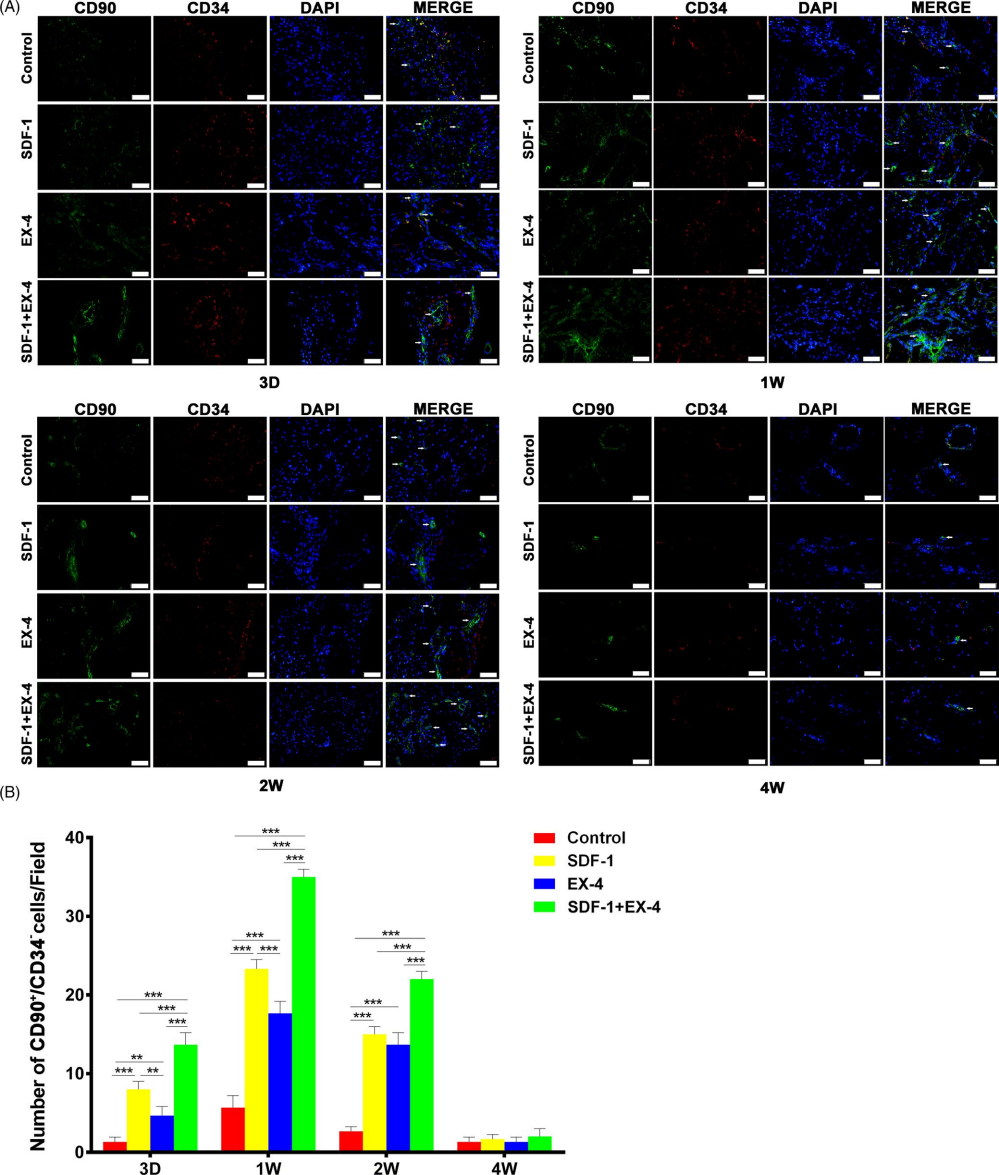
SDF‐1/EX‐4 cotherapy significantly promoted the migration of CD90^+^/CD34^‐^ cells in defect areas. A, Double immunofluorescence staining of CD34 (red) and CD90 (green) in the mandible sections at day 3, week 1, 2 and 4 post‐surgery (×400). Scale bar: 50 μm. White arrows, CD90^+^/CD34^‐^ cells. B, The number of CD90^+^/CD34^−^ cells (green) in SDF‐1+EX‐4 group was significantly larger than that in control, SDF‐1 or EX‐4 groups. The number of CD90^+^/CD34^−^ cells peaked at week 1, reduced at week 2 and hardly detected at week 4. ^**^ *P* < .01 and ^***^ *P* < .001

During the early healing process (day 3, week 1 and 2), the number of CD90^+^/CD34^‐^ cells in SDF‐1, EX‐4 or SDF‐1+EX‐4 groups significantly increased compared with control group (Figure 7A, B; *P* < .01). The number of CD90^+^/CD34^‐^ cells in SDF‐1+EX‐4 group was significantly larger than that in SDF‐1 or EX‐4 groups at three time points (*P* < .001). More CD90^+^/CD34^−^ cells were observed in SDF‐1 group than EX‐4 group at day 3 or week 1 (*P* < .01). The number of CD90^+^/CD34^−^ cells peaked at week 1 and decreased obviously at week 2. Unsurprisingly, few CD90^+^/CD34^−^ cells were seen in the defect areas at week 4.

### 3.7 SDF‐1/EX‐4 cotherapy enhanced the early osteoclastogenesis

**FIGURE 8:**
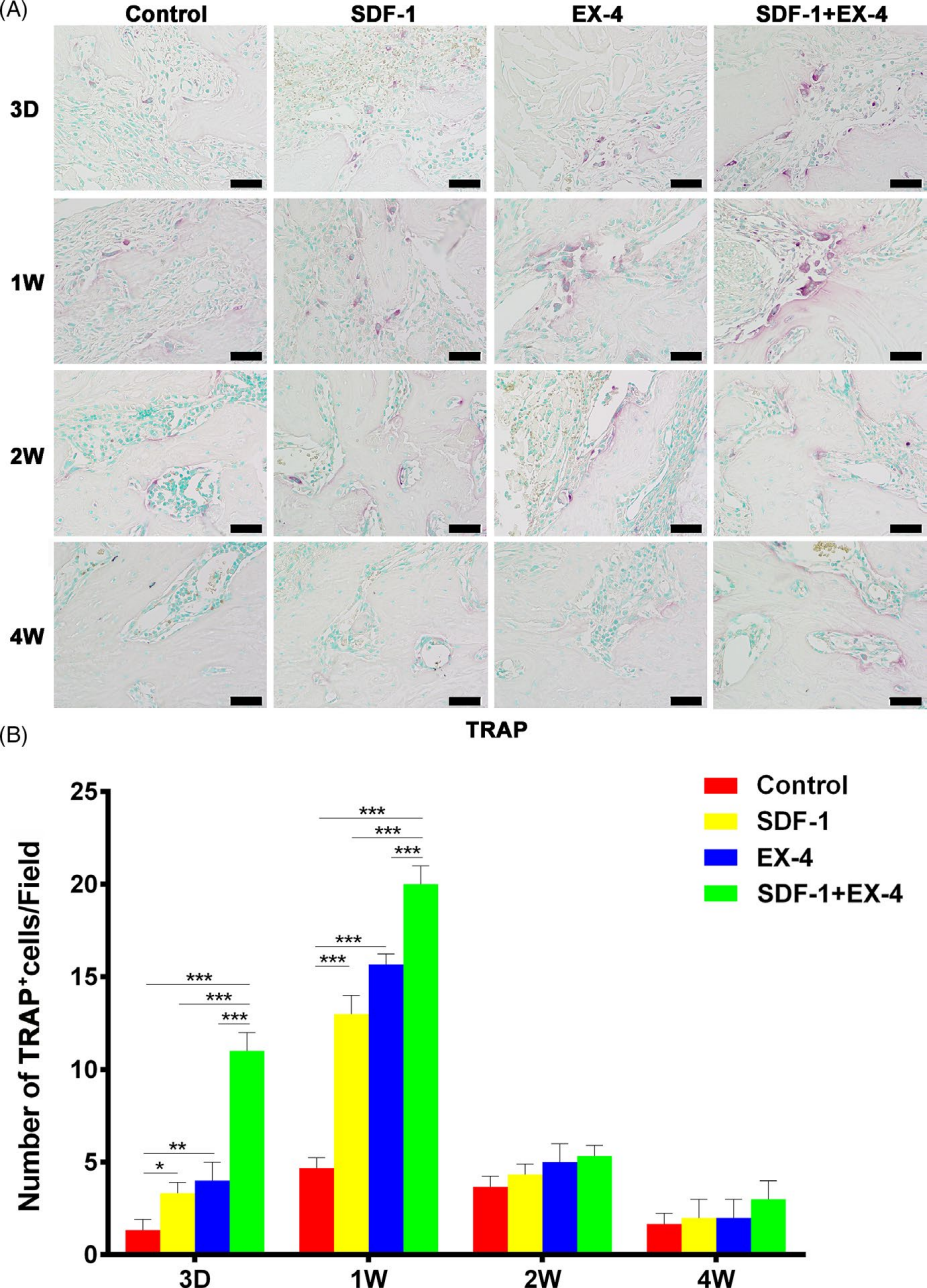
SDF‐1/EX‐4 cotherapy enhanced the early‐stage osteoclastogenesis. A, TRAP (red) staining in the mandible sections at day 3, week 1, 2 and 4 post‐surgery. The nucleus is green staining (×400). Scale bar: 50 μm. B, The number of TRAP^+^ cells in SDF‐1+EX‐4 group was significantly larger than that in control, SDF‐1 or EX‐4 groups. ^*^ *P* < .05, ^**^ *P* < .01 and ^***^ *P* < .001

SDF‐1, EX‐4 or SDF‐1+EX‐4 significantly increased the number of TRAP^+^ cells compared with control group at day 3 and week 1 (Figure 8A, B; *P* < .05). The number of TRAP^+^ cells in SDF‐1+EX‐4 group was two folds as much as that in SDF‐1 or EX‐4 groups at day 3 (*P* < .001). TRAP^+^ cells peaked at week 1, reduced obviously at week 2 and hardly detected at week 4.

### 3.8 SDF‐1/EX‐4 cotherapy promoted osteogenesis

**FIGURE 9:**
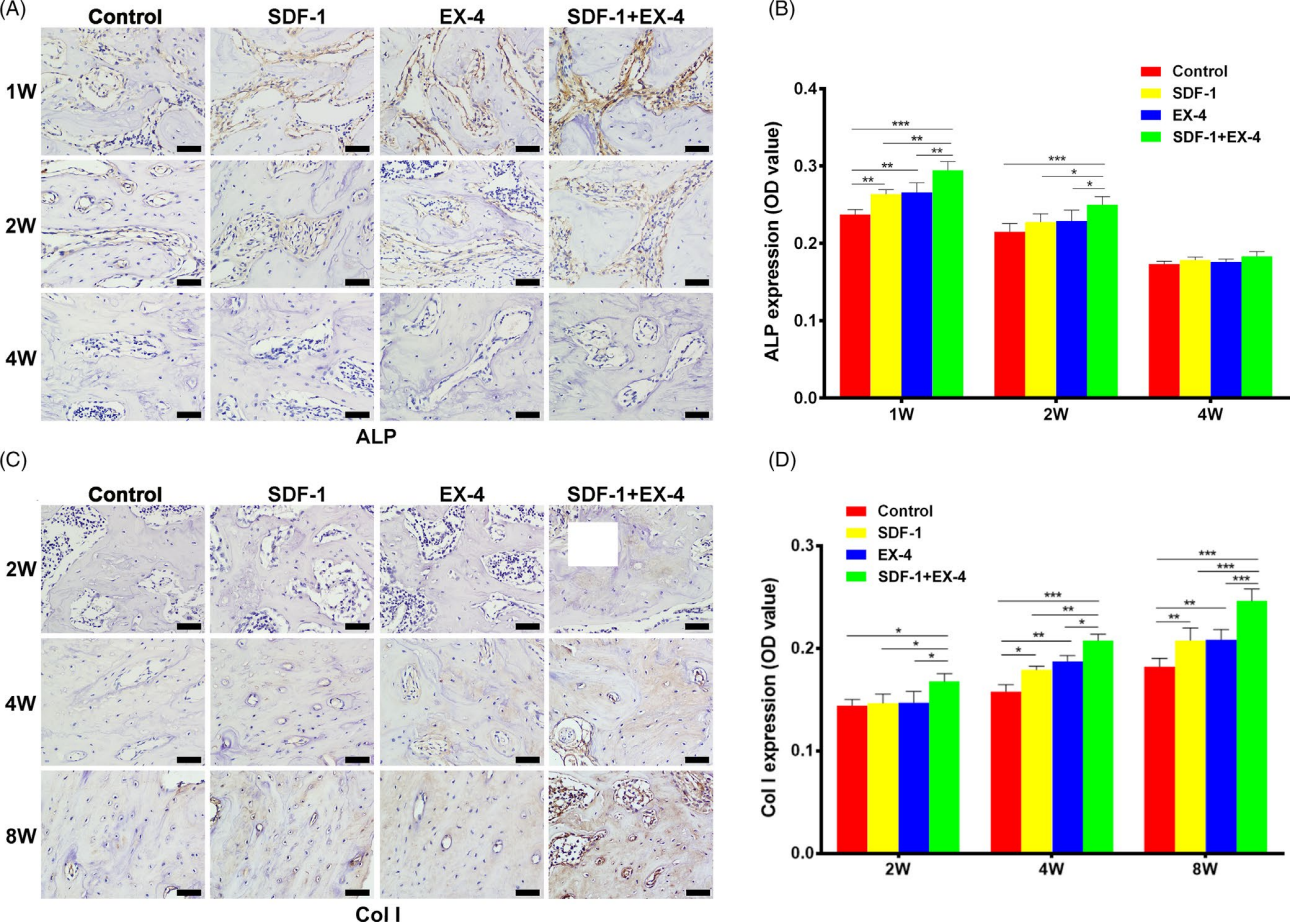
SDF‐1/EX‐4 cotherapy significantly promoted protein expression of osteogenesis‐related markers in regenerated tissues. A, Immunohistochemical staining of ALP (brown) at week 1, 2 and 4 (×400). Scale bar: 50 μm. B, Quantitative analyses of ALP expression in four groups. C, Immunohistochemical staining of Col I (brown) at week 2, 4 and 8 (×400). Scale bar: 50 μm. D, Quantitative analyses of Col I expression in four groups. ^*^ *P* < .05, ^**^ *P* < .01 and ^***^ *P* < .001

ALP expression was significantly increased in SDF‐1, EX‐4 or SDF‐1+EX‐4 groups than that in control group at week 1 (*P* < .01), while there was no significant difference between SDF‐1 and EX‐4 groups (*P* > .05; Figure 9A,B). ALP expression was reduced obviously at week 2 and was hardly detected in all four groups at week 4. The expression of Col I was significantly upregulated in SDF‐1+EX‐4 group compared with control, SDF‐1 or EX‐4 groups at week 2, 4 and 8 (*P* < .05; Figure 9C,D). At week 4 and 8, Col I expression in SDF‐1 or EX‐4 groups was significantly larger than that in control group (*P* < .05), while no significant difference was found between SDF‐1 and EX‐4 groups.

## 4 DISCUSSION

The purpose of this study was to evaluate the effect of cotherapy with a potent stromal cell homing factor SDF‐1 and an anti‐diabetic drug EX‐4 on periodontal bone regeneration. Firstly, we demonstrated that SDF‐1/EX‐4 cotherapy enhanced the proliferation, chemotaxis and osteogenic differentiation of PDLSCs in vitro. Secondly, periodontal bone regeneration was significantly promoted by topical application of SDF‐1 and systemic injection of EX‐4 in vivo.

SDF‐1 triggered promotion effects on MSC proliferation and migration were mainly mediated through SDF‐1/CXCR4 signalling pathway.^6^, ^7^, ^8^, ^10^ We previously demonstrated that SDF‐1 dose‐dependently enhanced proliferation and migration of PDLSCs in vitro.^6^ The recruitment of endogenous cells and promotion of periodontal tissue regeneration in vivo were reported by our team and other scholars.^9^, ^11^, ^12^ However, when applied alone in vivo, SDF‐1 does not accomplish ideal new bone formation. It is proposed that in vivo bone formation by SDF‐1 is induced by indirect effects such as the activation of cell trafficking, and not by the direct promotion of osteogenesis.^8^ Therefore, an osteopromotive drug needs to be combined with SDF‐1 to ensure the desired bone regenerative effect.

EX‐4, an anti‐diabetic agent, shares a 53% amino acid sequence homology with human GLP‐1.^25^ In addition to the hypoglycaemic function, EX‐4 also has positive effects on the proliferation and migration of MSCs.^20^, ^21^ A previous report demonstrated that EX‐4 promoted the proliferation of BMSCs in a time‐ and dose‐dependent manner.^21^ As for PDLSCs, our study was consistent with a recent report that EX‐4 has no beneficial effect on the proliferation of PDLSCs.^26^, ^27^ The migration of PDLSCs were promoted by EX‐4 in our study. More interestingly, SDF‐1 enhanced PDLSCs proliferative and chemotactic capabilities, and SDF‐1/EX‐4 cotherapy further strengthened the proliferation and chemotaxis effect. The additive effects suggested that EX‐4 may play an important role in SDF‐1‐mediated biological functions of PDLSCs.

Several pathways have been reported to be involved in cell proliferation and migration, including ERK and PI3K/AKT pathways.^28^, ^29^ ERK signalling pathway plays an important role in regulating SDF‐1 induced cellular functions.^29^, ^30^ Since ERK signalling pathway also plays a pivotal role in EX‐4 mediated adipose‐derived mesenchymal stem cells (ADSCs) and preosteoblast proliferation, here we investigated whether it was involved in the effect of SDF‐1/EX‐4 cotherapy on PDLSC proliferation.^20^ Both SDF‐1 and EX‐4 independently promoted the phosphorylation of ERK within a short time period, and SDF‐1/EX‐4 cotherapy further upregulated the phosphorylation of ERK. ERK signalling pathway inhibitor U0126 abolished SDF‐1/EX‐4 cotherapy induced PDLSC proliferation, which confirmed that ERK played an important role in PDLSC proliferation. However, ERK pathway was not involved in SDF‐1/EX‐4 cotherapy induced PDLSCs migration. PI3K/AKT pathway is a survival mediator that regulates various biological responses such as proliferation, apoptosis, and migration of MSCs.^28^, ^31^, ^32^ Previous studies demonstrated that SDF‐1‐ or EX‐4‐mediated chemotactic capability of MSCs was related to the activation of PI3K/AKT signalling pathway.^21^, ^33^ SDF‐1 and EX‐4 activated PI3K/AKT signalling pathway in our study, but the combination did not induce an additive effect.^34^ Further studies should be done to explore the mechanism underlying SDF‐1+EX‐4 mediated PDLSC migration.

EX‐4 could directly promote MSC osteogenesis according to recent studies.^22^, ^24^ Our results suggested that EX‐4 directly enhanced osteogenic differentiation of PDLSCs. When combined with SDF‐1, osteogenic differentiation was markedly enhanced compared with EX‐4 or SDF‐1 alone. ALP, BSP, OPN and OCN are important markers for assessing MSC osteogenic differentiation at different stages.^35^ ALP and BSP are early markers which reflect the degree of osteoblast osteogenic differentiation.^36^ OCN and OPN are late markers which suggest osteoblasts were matured.^37^ In this study, the gene expression levels of *ALP*, *BSP*, *OPN* and *OCN* were significantly upregulated with SDF‐1/EX‐4 cotherapy at different stages in vitro. A previous report suggested that EX‐4 promoted osteogenic differentiation of BMSCs by regulating Wnt/β‐catenin signalling pathway.^22^ EX‐4 also promoted osteogenic differentiation of PDLSCs in inflammatory microenvironment via inhibiting NF‐κB signalling pathway and activating Wnt/β‐catenin signalling pathway.^27^ Tamura M et al demonstrated that Wnt signalling regulated CXCL12 gene expression at the transcriptional level.^38^ Therefore, it is possible that Wnt/β‐catenin pathway is involved in osteogenic differentiation of PDLSCs induced by SDF‐1/EX‐4 cotherapy. The exact mechanisms will be explored by RNA sequencing technologies in our further study.

The potential of SDF‐1/EX‐4 cotherapy on periodontal bone regeneration was evaluated by topical application of SDF‐1 and systemic injection of EX‐4 in a rat periodontal bone defect model. We previously reported that local delivery of SDF‐1 recruited host‐derived MSCs and promoted the quality and quantity of regenerated bones.^11^ It has been reported that EX‐4 induced bone formation in T2DM mice, aged ovariectomized (OVX) rats and hindlimb‐unloading rats, although the underlying mechanisms are not completely understood.^39^ The present analysis of the effect of EX‐4 on bone remodelling focus on the mode of action including blood supply to bone, the hormone regulation and MSC osteogenic differentiation. Therefore, we applied EX‐4 systemically based on the previous data in the animal experiments. Similar to our previous findings, SDF‐1 group exhibited higher indexes of BV/TV, BS/TV and Tb.Th and a lower index of Tb.Sp compared with control group according to micro‐CT images. However, no satisfactory periodontal bone regeneration could be observed at week 8 post‐surgery with SDF‐1 or EX‐4 alone. In contrast, the combination of SDF‐1 and EX‐4 significantly accelerated the regeneration of periodontal bone tissues. The enhancement of new bone quantity and density via SDF‐1 was obviously promoted by cotherapy with EX‐4, indicating that SDF‐1/EX‐4 cotherapy has significantly additive effect on periodontal bone regeneration. To further investigate the underlying mechanisms, endogenous cell migration, CXCR4 expression, early osteoclastogenesis and osteogenesis were evaluated in vivo.

Adult stem cells expressing CD90, a cell surface marker of stem and progenitor cells, possess high potential to undergo osteogenic differentiation.^40^ CD34 is widely used as a marker for haematopoietic cells. CD90^+^/CD34^−^ cells were considered to be non‐haematopoietic stromal cells, which possess osteogenic differentiation potential. Our results showed that there was more CD90^+^/CD34^−^ stromal cell mobilization after topical application of SDF‐1 alone. It is well known that SDF‐1 and CXCR4 interaction is responsible for recruiting MSCs and osteogenic progenitor cells together with the other types of cells to the injured sites.^10^, ^11^ Our results indicated that SDF‐1 increased the number of CXCR4^+^ cells during the early stage of defect repair. SDF‐1 may enhance new bone formation by recruitment of endogenous cells.^11^, ^41^ Interestingly, after treatment with EX‐4, MSC displayed a higher proliferative capacity, increased CXCR4 expression and enhanced migration evidenced by the transwell and wound‐healing assays in vitro.^21^ Similarly, EX‐4 promoted the proliferation and expression of CXCR4 in the ADSCs, increased the secretion of SDF‐1 in cardiomyocytes and enhanced the migration of the ADSCs by activating the PI3K/Akt pathway.^33^ These studies illustrated that EX‐4 could increase the expression of CXCR4 in MSCs, improve their biological functions, especially their proliferation and migration. In addition, EX‐4 increased the numbers of BMSCs and osteoblasts migration to the bone surface in OVX mice, probably by increasing TGF‐β1 expression.^42^ In the present study, we first explored whether EX‐4 could recruit endogenous cells to periodontal defects in vivo. Our data showed that EX‐4 promoted CD90^+^/CD34^‐^ stromal cell and CXCR4^+^ cell migration to defect area. Moreover, EX‐4 significantly enhanced SDF‐1‐induced recruitment of the host cells. It's worth noting that SDF‐1 displayed more stronger potential to mobilize stem cells than EX‐4 in vitro and in vivo. However, the newly formed bones in SDF‐1 group were similar to EX‐4 group in periodontal defect in vivo. The findings suggest that regenerative strategies targeted at recruiting cells are not sufficient for successful periodontal bone regeneration. Orchestrating their differentiation into tissue‐forming cells in the next stage is extremely important.

Bone remodelling depends on the coordination of bone resorption by osteoclasts and bone matrix synthesis by osteoblasts, thus osteoclasts play a vital role in the process of bone repair.^43^, ^44^ At the early stage of healing, osteoclasts create resorption pits, growth factors such as TGF‐β are released from the bone matrix. The growth factors may recruit MSCs and osteoblast progenitors, promote these cells proliferation, differentiation with matrix formation and mineralization to fill resorbed bone area.^44^, ^45^ We investigated the effects of SDF‐1 or/and EX‐4 on osteoclastogenesis in vivo and our TRAP staining results showed that both SDF‐1 and EX‐4 significantly increased the number of osteoclasts in the defect area from day 3, suggesting that SDF‐1 and EX‐4 initiated early osteoclastogenic events. Our results are consistent with previous studies which showed SDF‐1 promoted chemotactic recruitment of pre‐osteoclasts through CXCR4 interaction. Following recruitment, SDF‐1 also acted indirectly through regulation of matrix metalloprotease (MMP, a matrix‐degrading enzyme essential for pre‐osteoclasts) but as osteoclast maturation took place, SDF‐1 was not involved.^46^, ^47^ These findings suggested that SDF‐1 promoted early (but not later) stages of osteoclast development and stimulated TRAP activity in early osteoclastogenic effects. However, previous investigations of EX‐4 on osteoclasts were conflicting. EX‐4 decreased the number of osteoclasts on the surface of trabecular bone after administration of EX‐4 to aged OVX rats for 16 weeks.^48^ In contrast, Pereira et al found a slight increase of osteoclast number and osteoclastic surface with EX‐4 in OVX mice in vivo.^49^ The reason for the discrepancy is unclear and may involve differences in experimental animals and the duration of EX‐4 therapy. Our results showed that the number of osteoclasts increased significantly in SDF‐1+EX‐4 group, indicating more active bone metabolism with this cotherapy manner. Together, SDF‐1+EX‐4 may upregulate old bone resorption to provide space for new bone formation by chemoattracting pre‐osteoclasts and initiating early osteoclastogenic events. ALP expression indicates extracellular matrix progressing into the mineralization phase.^36^ Col I expression reflects the mineralization of osteoblasts. Our results showed that the expression of ALP and Col I were enhanced by SDF‐1/EX‐4 cotherapy, which suggested the combination of SDF‐1 and EX‐4 promoted the process of osteogenesis by accelerating osteoblast differentiation at an early stage and mineralization at a late age, thus enhanced bone formation.

Further, rat periodontal defect model results indicated that SDF‐1 or EX‐4 alone enhanced periodontal bone formation. In addition, EX‐4 further enhanced SDF‐1 induced periodontal bone regeneration in vivo. There are some potential explanations for this synergetic effect. Firstly, MSCs and bone marrow‐derived osteoblast progenitor cells might be engrafted from the circulating blood towards the periodontal defects via SDF‐1 or EX‐4.^11^, ^22^, ^42^ In our study, more host stromal cells were recruited in SDF‐1+EX‐4 group. The recruited cells not only exerted their multidirectional differentiation ability, but also secreted a variety of growth factors to promote tissue healing.^42^, ^45^ Secondly, SDF‐1 and EX‐4 can maintain the cellular survival and growth of the recruited MSCs or osteoblast progenitor cells until osteogenic differentiation is elicited. Thirdly, SDF‐1 and EX‐4 facilitated the engrafted cell differentiate to exert their osteogenic effects to promote bone regeneration. Moreover, both SDF‐1 and EX‐4 have pro‐angiogenesis and anti‐inflammatory effects during chronic inflammatory disease.^27^, ^50^ The improvement of angiogenesis and inflammatory conditions in periodontal defects may provide a suitable microenvironment for new bone formation. However, which mechanism plays the key role in the periodontal bone regeneration remains to be elucidated.

In conclusion, cotherapy with 50 ng/mL SDF‐1 and 10 nmol/L EX‐4 enhanced the proliferation, chemotaxis and osteogenic differentiation of PDLSCs in vitro. Moreover, SDF‐1/EX‐4 cotherapy enhanced the recruitment of MSCs, induced early osteoclastogenesis, promoted the expression of osteogenic proteins in newly formed bone tissues and improved the quantity and quality of regenerated bones in vivo. Therefore, SDF‐1/EX‐4 cotherapy provides a new strategic option for in situ periodontal bone regeneration.

## CONFLICT OF INTEREST

The authors declare that there is no conflict of interests.

## AUTHOR CONTRIBUTIONS

QYL performed the experiments and wrote the manuscript. LQD performed the experiments and reviewed manuscript. RZ performed the experiments. WYK analysed the data and prepared figures. SHG designed the study and revised the manuscript.

## Supporting information

Supplementary Material

## Data Availability

The data that support the findings of this study are available from the corresponding author upon reasonable request.
