# Supplementary material for: Stromal cell‐derived factor‐1/Exendin‐4 cotherapy facilitates the proliferation, migration and osteogenic differentiation of human periodontal ligament stem cells in vitro and promotes periodontal bone regeneration in vivo
Source: Cell Prolif. 2021 Jan 29;54(3):e12997. doi: 10.1111/cpr.12997 (PMC7941242; doi:10.1111/cpr.12997)
Supplement: Supplementary file 1 — Supplementary Material [file CPR-54-e12997-s001.docx]

**Stromal cell-derived factor-1/Exendin-4 cotherapy facilitates the proliferation, migration and osteogenic differentiation of human periodontal ligament stem cells *in vitro* and promotes periodontal bone regeneration *in vivo***

Qianyu Liang,^1, #^ Lingqian Du,^2, #^ Rui Zhang,^1,3^ Wenyan Kang,^1^ Shaohua Ge ^1*^

*^1^Department of Periodontology, School and Hospital of Stomatology, Cheeloo College of Medicine, Shandong University & Shandong Key Laboratory of Oral Tissue Regeneration & Shandong Engineering Laboratory for Dental Materials and Oral Tissue Regeneration, Jinan Shandong 250012, China*

^2^*Department of Stomatology, The Second Hospital, Cheeloo College of Medicine, Shandong University, Jinan Shandong 250033, China*

*^3^Department of Endodontics, Hospital of stomatology, Zunyi Medical University, Zunyi Guizhou 563000, China*

**Correspondence**

Shaohua Ge, No. 44-1 Wenhua Road West, Jinan, China, E-mail address: shaohuage@sdu.edu.cn

Qianyu Liang and Lingqian Du contributed equally to this work and they should be regarded as co-first author.

**S1 MATERIALS AND METHODS**

**S1.1 Isolation and characterization of human PDLSCs**

Periodontal ligament (PDL) tissues from the middle third of the root surface were collected and digested with 3 mg/mL collagenase I (Sigma Aldrich, St Louis, MO, USA) and 4 mg/mL dispase II (Invitrogen, Carlsbad, CA, USA) for 40 minutes at 37°C. The single-cell suspension of primary cells was filtered through a 70 μm cell strainer (BD Biosciences, Bedford, MA, USA), seeded into 10-cm petri dishes (Corning, Corning, NY, USA) at a density of 60 cells/cm^2^ and cultured in media containing Dulbecco’s modified Eagle’s medium (DMEM, Hyclone, Logan, UT, USA) with 20% fetal bovine serum (FBS, BioInd, Kibbutz, Israel) and 1% antibiotics (100 U/mL of penicillin, 100 mg/mL of streptomycin, Sigma Aldrich). The passaged PDLSCs were cultured in DMEM with 10% FBS (basic media) and PDLSCs at passages 4-6 were used in the following experiments. For osteogenic differentiation, PDLSCs were cultured with osteogenic inductive media [OM, basic media with 10^-8^ mol/L dexamethasone (Solarbio, Beijing, China), 50 mg/L ascorbic acid and 10 mmol/L β-glycerophosphate (Sigma Aldrich)] for 28 days, the mineral deposition was stained with Alizarin Red S (pH 4.3, Sigma Aldrich). For adipogenic differentiation, PDLSCs were cultured with adipogenic inductive media [basic media with 0.5 mmol/L 3-isobutylethylxanthine, 0.5 μmol/L hydrocortisone, 2 mmol/L insulin, and 60 μM indomethacin (Solarbio)] for 28 days, the presence of lipid drops was detected by staining with Oil Red O (Solarbio).

**S1.2 Immunofluorescence assay of CXCR4**

PDLSCs were cultured in basic media for 24 hours. Cells were fixed with 4% paraformaldehyde (Sigma Aldrich) and ruptured of cell membranes with 1% Triton-X (Solarbio). After blocking with 1% bovine serum albumin (BSA, AMRESCO, Solon, OH, USA), the cells were incubated with rabbit anit-CXCR4 antibody (1:200, ab124824, Abcam, Cambridge, MA, USA) at 4°C overnight. Cells were respectively incubated with goat anti-rabbit IgG (1:200, SA00013-4, Proteintech, Rosemont, IL, USA) in the dark for 1 hour. Nuclei were visualized with 2-(4-amidinophenyl)-6-indolecarbamidine dihydrochloride (DAPI, Solarbio) and mounted. The images were photographed under a fluorescence microscope (Olympus Corporation, Shinjuku-ku,Tokyo, Japan) (N=6).
**S1.3 Cell proliferation assay**

PDLSCs were seeded in 96-well plates at a density of 3×10^3^ cells/well and cultured in basic media. After 24 hours, cells were cultured in maintenance media (DMEM with 2% FBS) or maintenance media with recombinant SDF-1 (Peprotech, Rocky Hill, NJ, USA) (10, 50, 100 or 200 ng/mL) or Exendin-4 (EX-4, R&D systems, Minneapolis, MN, USA) (5, 10, 20 or 50 nmol/L) for 3 days. Cell-counting kit-8 (CCK8, Dojindo Laboratories, Kumamoto, Japan) solution was added. The absorbance was detected by a microplate reader (SPECTROstar Nano, BMG Labtech, Offenburg, Germany) at a wavelength of 450 nm. Experiments were performed in sextuplicate (N=6).

**S1.4 Cell migration assay**

The migratory effect of SDF-1 or EX-4 on PDLSCs was evaluated by an 8 µm transwell chamber (Corning). PDLSCs were seeded onto the upper chamber at a density of 5×10^4^ cells/well and cultured in maintenance media. The lower plates were supplemented in 500 µL maintenance media with SDF-1 (10, 50, 100 or 200 ng/mL) or EX-4 (5, 10, 20 or 50 nmol/L). 500 µL maintenance media served as a negative control (NC) and 500 µL basic media served as a positive control (PC). After 20 hours, cells that had migrated through the membrane were fixed with 4% paraformaldehyde and stained with 0.1% crystal violet (Solarbio). Then cells were observed under a microscope and six randomly selected high-power microscopic fields (×200) per filter were counted. Experiments were performed in triplicate (N=3).

**S1.5 Detection of the optimal effect concentration of SDF-1/EX-4 cotherapy on PDLSCs**

Cell proliferation assay and cell migration assay were performed to detect the optimal effect concentration of SDF-1/EX-4 cotherapy on PDLSCs.

**S1.5.1** **Cell proliferation assay**

PDLSCs were seeded in 96-well plates at a density of 3×10^3^ cells/well and cultured in basic media. After 24 hours, cells were cultured in maintenance media, maintenance media with SDF-1 (10, 50 or 100 ng/mL) and 10 nmol/L EX-4 for 3 days. And the absorbance was detected according to S1.3. Experiments were performed in sextuplicate (N=6).

**S1.5.2** **Cell migration assay**

PDLSCs were seeded onto upper chamber at a density of 5×10^4^ cells/well and cultured in maintenance media. The lower plates were supplemented in 500 µL maintenance media with SDF-1 (10, 50 or 100 ng/mL) and 10 nmol/L EX-4. 500 µL maintenance media served as a NC and 500 µL basic media served as a PC. And the cells that had migrated were observed according to S1.4. Experiments were performed in triplicate (N=3).

**S2 RESULTS**

**S2.1 Characteristics of PDLSCs and expression of CXCR4**

Human PDLSCs were isolated form normal PDL tissues and exhibited spindle-shaped fibroblast-like morphology under microscope (Figure S1A). After induction for 28 days, PDLSCs displayed mineralized nodules (Figure S1B) and lipid droplets (Figure S1C). Cultured PDLSCs exhibited positive staining for CXCR4 (Figure S1D).

**S2.2 SDF-1 enhanced the proliferation of PDLSCs**

10, 50 and 100 ng/mL SDF-1 significantly promoted the proliferation of PDLSCs compared with control group at day 3 (*P*<.001) (Figure S2A). 200 ng/mL SDF-1, 5, 10, 20 and 50 nmol/L EX-4 presented no significant effect on PDLSC proliferation compared with control group (*P*>.05) (Figure S2B). The results showed that 10, 50 and 100 ng/mL SDF-1 dramatically enhanced the proliferation of PDLSCs.

**S2.3 SDF-1 or EX-4 enhanced the migration of PDLSCs**

**S2.3.1 SDF-1 enhanced the migration of PDLSCs**

50 ng/mL SDF-1 significantly enhanced the migration capacity of PDLSCs compared with NC, 10, 100 and 200 ng/mL SDF-1 (46.2±2.04 *vs* 14.2±3.31, 34.7±2.73, 34.3±3.14, 26.5±2.95 cells/field, *P*<.001) (Figure S3A). Recruited cells in 50 ng/mL SDF-1 group were less than PC (46.2±2.04 *vs* 75.3±3.39 cells/field, *P*<.001). 10, 100 and 200 ng/mL SDF-1 also promoted PDLSC migration compared with NC (*P*<.001). These results indicated that SDF-1 enhanced PDLSC migration. Basing on cell proliferation and migration results, 10, 50, and 100 ng/mL SDF-1 were selected for the subsequent experiments.

**S2.3.2 EX-4 enhanced the migration of PDLSCs**

10 nmol/L EX-4 significantly enhanced the migration capacity of PDLSCs compared with NC, 5, 20 and 50 nmol/L EX-4 (34.0±2.97 *vs* 12.3±2.81, 25.3±1.03, 24.6±1.75, 20.0±1.41 cells/field, *P*<.001) (Figure S3B). Recruited cells in 10 nmol/L EX-4 group were less than PC (34.0±2.97 *vs* 76.3±5.20 cells/field, *P*<.001). 5, 20 and 50 nmol/L EX-4 also promoted PDLSC migration compared with NC (*P*<.001). The results indicated that EX-4 enhanced PDLSC migration. Basing on cell proliferation and migration results, 10 nmol/L EX-4 were selected for the subsequent experiments.

**S2.4** **SDF-1/EX-4 cotherapy enhanced the proliferation of PDLSCs**

SDF-1/EX-4 cotherapy significantly promoted the proliferation of PDLSCs compared with control group at day 3 (*P*<.001) (Figure S4A). 50 ng/mL SDF-1 and 10 nmol/L EX-4 cotherapy exhibited excellent promotion effect compared with the other groups (*P*<.001). The results showed that 50 ng/mL SDF-1 and 10 nmol/L EX-4 cotherapy dramatically enhanced the proliferation of PDLSCs.

**S2.5** **SDF-1/EX-4 cotherapy enhanced the migration of PDLSCs**

50 ng/mL SDF-1 and 10 nmol/L EX-4 cotherapy significantly enhanced the migration capacity of PDLSCs compared with NC, 10 or 100 ng/mL SDF-1 cotherapy groups (54.7±2.42 *vs* 14.2±3.31, 44.8±2.86, 45.3±2.27 cells/field, *P*<.001) (Figure S4B, C). 10 or 100 ng/mL SDF-1 cotherapy with 10 nmol/L EX-4 also promoted PDLSC migration compared with NC (*P*<.001). Recruited cells in 50 ng/mL SDF-1 and 10 nmol/L EX-4 cotherapy group were less than PC (54.7±2.42 *vs* 75.3±3.39 cells/field, *P*<.001). These results indicated that 50 ng/mL SDF-1 and 10 nmol/L EX-4 cotherapy dramatically enhanced PDLSC migration. Basing on cell proliferation and migration results, 50 ng/mL SDF-1 and 10 nmol/L EX-4 were selected for the subsequent experiments.

**FIGURE LEGENDS**

**
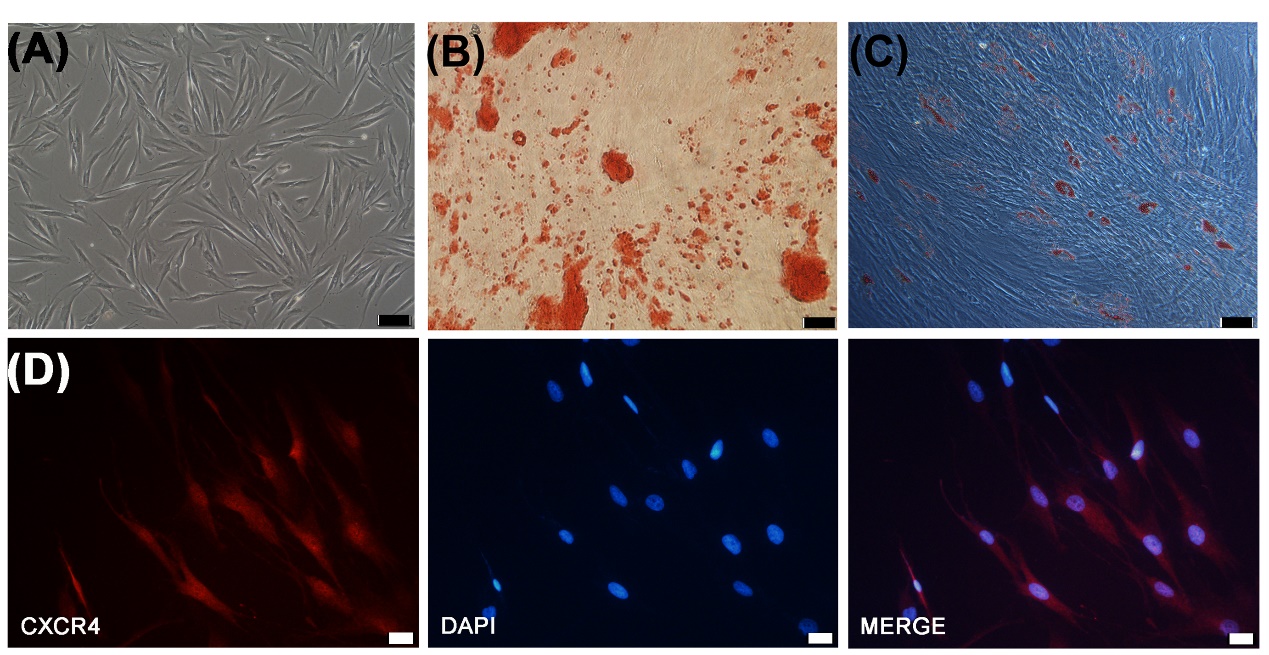
**

**FIGURE S1** Characteristics of PDLSCs and expression of CXCR4. A, PDLSCs exhibited typical fibroblast-like morphology (×100). Scale bar: 100 μm. B, Alizarin Red S staining of PDLSCs at day 28 (×100). Scale bar: 100 μm. C, Oil red O staining of PDLSCs at day 28 (×100). Scale bar: 100 μm. D, PDLSCs exhibited positive staining for CXCR4. CXCR4 exhibited red fluorescence and the nucleus showed blue fluorescence (×200). Scale bar: 20 μm.


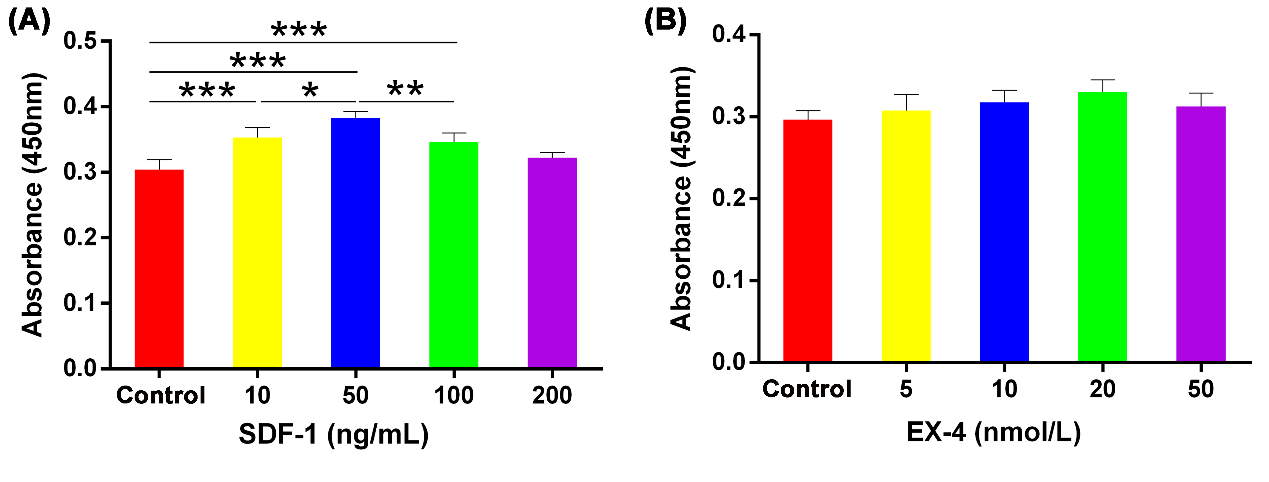


**FIGURE S2** SDF-1 enhanced the proliferation of PDLSCs. A, 10, 50 and 100 ng/mL SDF-1 significantly promoted the proliferation of PDLSCs compared with control group. B, EX-4 has no significant effect on the proliferation of PDLSCs. ^*^*P*<.05, ^**^*P*<.01 and ^***^*P*<.001


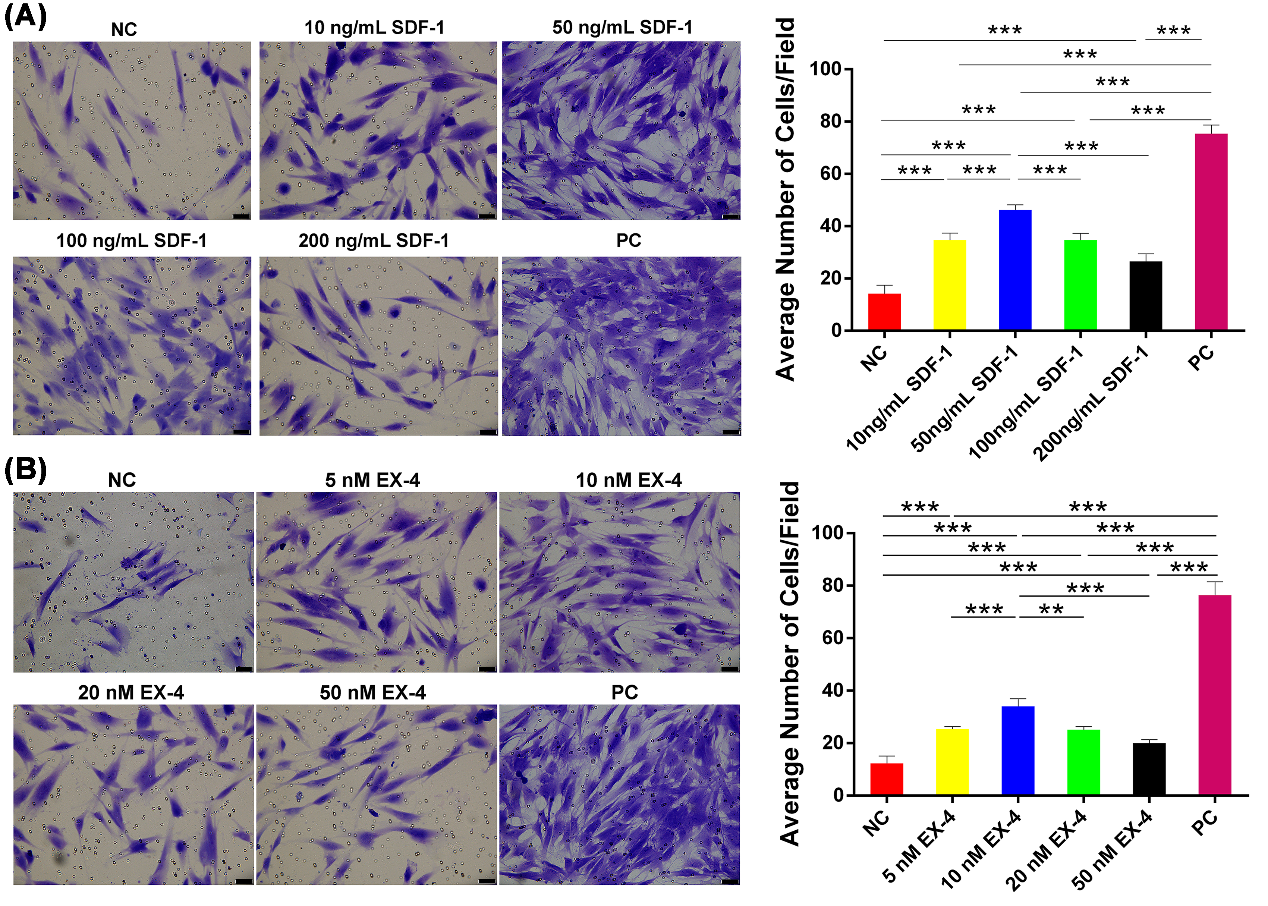


**FIGURE S3** SDF-1 and EX-4 enhanced the migration of PDLSCs. A, 10, 50, 100 and 200 ng/mL SDF-1 significantly promoted the migration of PDLSCs compared with NC (×200). Scale bar: 50 μm. B, 5, 10, 20 and 50 nmol/L EX-4 significantly promoted the migration of PDLSCs compared with NC (×200). Scale bar: 50 μm. NC, negative control. PC, positive control. ^**^*P*<.01 and ^***^*P*<.001.


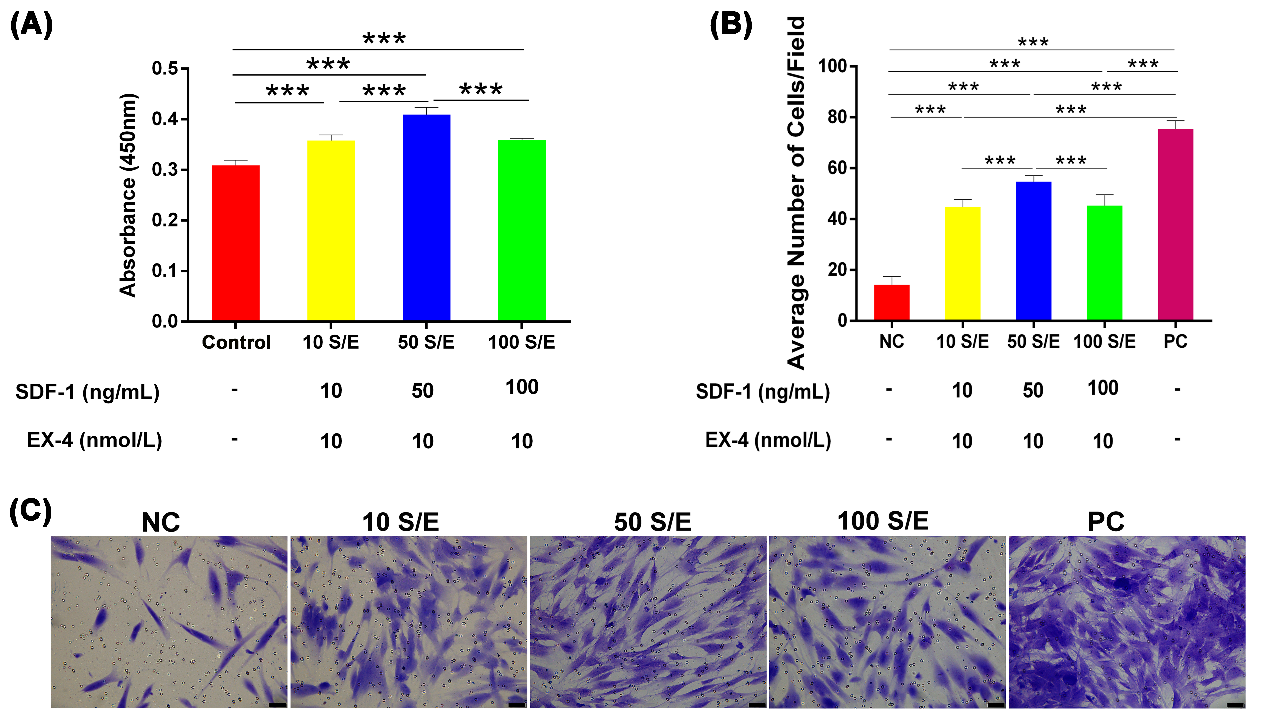


**FIGURE S4** SDF-1/EX-4 cotherapy enhanced the proliferation and migration of PDLSCs. A, 50 ng/mL SDF-1 and 10 nmol/L EX-4 cotherapy significantly promoted the proliferation of PDLSCs compared with control, 10 or 100 ng/mL SDF-1 cotherapy groups. B, 50 ng/mL SDF-1 and 10 nmol/L EX-4 cotherapy significantly enhanced the migration capacity of PDLSCs compared with NC, 10 or 100 ng/mL SDF-1 cotherapy groups. C, Crystal violet staining showed the cells that migrated to the undersurface of the membrane in different groups (×200). Scale bar: 50 μm. 10, 50 and 100 S/E represented 10, 50 or 100 ng/mL SDF-1+10 nmol/L EX-4. NC, negative control. PC, positive control. ^***^*P*<.001.
